# Creatinine-to-cystatin C ratio as a marker of sarcopenia for identifying osteoporosis in male patients with type 2 diabetes mellitus

**DOI:** 10.1186/s12891-022-05636-8

**Published:** 2022-07-14

**Authors:** Huifan Dai, Jing Xu

**Affiliations:** 1 Department of Endocrinology, The Second Affiliated Hospital and Yuying Children’s Hospital of Wenzhou Medical University, Wenzhou, China; 2 Department of Endocrinology, The Second Affiliated Hospital and Yuying Children’s Hospital of Wenzhou Medical University, Lucheng District, Wenzhou, Zhejiang Province People’s Republic of China

**Keywords:** Type2 diabetes, Osteoporosis, Bone mineral density

## Abstract

**Background:**

Type 2 diabetes mellitus (T2DM) is associated with the increased incidence rate of sarcopenia and osteoporosis. Serum creatinine-to-cystatin C ratio (CCR) is a novel and simple tool which can be used as an index of sarcopenia. This study aims to investigate the association between CCR and osteoporosis as well as bone mineral density (BMD) in T2DM patients.

**Methods:**

Four hundred eighteen T2DM patients were recruited, including 166 females and 252 males. General information, BMD data and laboratory data were collected. The correlation between CCR, BMD, bone metabolism markers and osteoporosis was explored by spearman correlation, receiver-operating characteristic (ROC) curve analysis and multiple regression analysis.

**Results:**

Spearman correlation analysis showed that there was a positive correlation between CCR and BMD as well as serum calcium in male patients (*r* = 0.181–0.381, *P* < 0.01), but such correlation was not found in the female group. In multivariate regression analysis, it was found that there was a significant correlation between CCR and BMD of total lumbar spine, hip as well as femoral neck in male patients. ROC curve showed that the optimal cut-off value of CCR for predicting osteoporosis in male patients was 6.73 with the sensitivity of 88% and specificity of 63%.

**Conclusion:**

In male T2DM patients, CCR was negatively correlated with osteoporosis and positively correlated with BMD.

## Introduction

Fractures and osteoporosis show a rising trend in the elderly, which can result in disability, decline in quality of life, and even death [1]. Patients with T2DM are generally more likely to suffer from fracture than the general population [2, 3]. Previous studies have analyzed and reported risk factor associated with osteoporosis in the general population [4]. However, it is important to explore the risk factors for osteoporosis, which may differ from those with T2DM due to metabolic disorders.

Sarcopenia, a geriatric disease characterized by a progressive loss of skeletal muscle mass and loss of muscle function, constitutes a rising, often undiagnosed health problem. The relationship between the sarcopenia and osteoporosis in chronic obstructive pulmonary disease [5], chronic kidney disease [6], primary biliary cholangitis [7] has been well developed. It is also confirmed that skeletal muscle mass is independently associated with osteoporosis in patients with T2DM [8]. However, traditional skeletal muscle mass inspection methods, such as dual-energy-X-ray absorption method, are complex and expensive.

Serum creatinine is a metabolic waste produced by skeletal muscle. Cystatin C can be produced by nucleated cells in the body at a constant production rate, which can only be removed through glomerular filtration. Some studies have supported that the creatinine-to-cystatin C ratio (CCR) can be used as a biomarker of skeletal muscle mass. Takafumi et al. suggested that CCR is an inexpensive and simple method to detect sarcopenia in T2DM patients [9]. However, whether this ratio can be used as a predictor of osteoporosis in `T2DM has not been studied. Therefore, this study aims to explore the relationship between CCR, osteoporosis and BMD in patients with T2DM.

## Methods

### Subjects

The study included 418 T2DM patients (age > 50 years old) in China. The subjects were evaluated or treated for T2DM at the Second Affiliated Hospital of Wenzhou Medical University and Yuying Children's Hospital from January 2020 to March 2021. Because serum cystatin C does not increase in those with severely impaired kidney function while serum creatinine dose increase [10, 11], we selected patients with normal renal function (glomerular filtration rate > 60 ml/min, creatine ≤ 115µmmol/L). Exclusion criteria included (a) malignant tumors and severe heart or liver diseases; (b) diagnosis of adrenal, gonadal, parathyroid, pituitary and thyroid diseases; (c) long-term use of calcium, vitamin D, or other drugs that influence bone metabolism for more than three months; (d) patients who were bedridden for a long time; and (e) patients who lacked available information. This study was approved by the Ethics Committee of the Second Affiliated Hospital of Wenzhou Medical University (No. LCKY2020-03, date: January 2020), and the written informed consent of all participants obtained according to the Declaration of Helsinki.

### Health history and clinical assessment

Their height and weight without wearing shoes were measured. BMI was calculated by dividing weight (Kg) by the square of height (m^2^). The duration of T2DM was calculated in years, which ranged from the diagnosis of T2DM in medical record of patient's to our BMD measurements and blood tests. Smoking and drinking history were considered as never or ever.

### Biochemical parameters

Serum samples were collected at 6 am after fasting at night (at least 8 h). Cobas c702 chemistry autoanalyzer (Roche Diagnostics, Switzerland) methods were used to measure serum lipid metabolism indicators, including high density lipoprotein cholesterol (HDL-C), total cholesterol (TC), low density lipoprotein cholesterol (LDL-C), triglyceride (TG); glucose metabolism indicators, including (FBG) fasting blood glucose, (HbA1c) glycosylated hemoglobin; Bone metabolism indicators, including β-CTX, PINP, 25-hydroxy-vitamin (25(OH)D) and parathyroid hormone (PTH); other laboratory markers such as creatinine, calcium, uric and albumin. Serum cystatin C concentrations were detected using an immunoturbidimetric technique with the Modular Analytics Cobas 6000 analyzer (Roche Diagnostics, Switzerland). The CCR was then calculated as the serum creatine concentration (mg/L) divided by the cystatin C concentration (mg/L).

### BMD measurement

Whole-body dual-energy X-ray absorptiometry (DXA; USA, Hologic Discovery) was used to measure body fat content, trunk muscle mass and BMD of the lumbar spine, femur neck, total hip and skeletal muscle mass index (ASMI). The calculation formula of ASMI is as follows: appendicular skeletal muscle mass (ASM)/height^2^ [12]. T scores was calculated by using the DXA database [13]. Osteoporosis, osteopenia, and normal bone mass was determined based on the following T-scores: ≤  − 2.5, − 2.5 to 1.0, and >  − 1.0, respectively, in the total hip, femoral neck, or lumbar area [13].

### Statistical analysis

The proportion was used to describe categorical variables and mean ± standard deviation to describe continuous variables. Intergroup differences were evaluated using one-way analysis of variance for normally distributed variables, using the Mann–Whitney U test for non-normally distributed variables, and using the chi-squared test for categorical variables. The BMD as well as incident of osteoporosis was different between genders; as a result, male and female were independently analyzed. Spearman coefficient was used to analyze CCR, BMD and bone metabolism index. Each parameter was selected for which *P* value < 0.05 in initial univariate results, age, diabetic duration, BMI, smoking, drinking, TC, TG, HDL-C, LDL-C, albumin, uric, HbA1c, FBG and CCR for the multivariate analyses for the correlation between BMD of femoral neck, lumbar spine, hip and other variables. Odds ratios (ORs) and 95% confidence intervals (CIs) were calculated by Logistic analysis. We plotted the receiver operating characteristic (ROC) curve and calculated the area under the curve (AUC) to assess the clinical value of CCR in predicting osteoporosis. In order to make CCR significantly different between osteoporosis group and normal group, with α = 0.05, two-tailed and a power of 80%, we needed at least 33 patients per group.

## Results

### Basic characteristics

Patient characteristics are shown in Table 1. The retrospective study included 418 T2DM patients with a BMI of 24.5 ± 3.2 kg/m^2^ and an average age of 60.8 ± 7.3 years old. The BMD of the hip, femur neck and lumbar spine were higher in males (1.007 vs 0.903, 0.941 vs 0.826, 1.194 vs 1.012, all *P* < 0.01) compared with females. The levels of HDL-c, FBG, HbA1c, LDL-c, PINP, β-CTX and the incidence of osteoporosis in males were significantly lower than those in females. The levels of creatinine, cystatin C, CCR and ASM/height^2^ were significantly higher in males than those in females.

**Table 1:** Patient characteristics, stratified by sex

	Total patients (*n* = 418)	male patients (*n* = 252)	female patients (*n* = 166)	P
Age, years	60.8 ± 7.3	59.8 ± 7.4	62.2 ± 6.9	0.001
Diabetic duration, years	9.2 ± 7.1	8.5 ± 6.9	10.2 ± 7.3	0.019
Systolic blood pressure, mmHg	142.7 ± 25.1	141.9 ± 24.3	143.9 ± 26.4	0.427
Diastolic blood pressure, mmHg	85.2 ± 41.7	87.4 ± 53.3	81.9 ± 7.6	0.186
Smoking (current or ever)	24.4%	39.7%	1.2%	< 0.001
Alcohol consumption (current or ever)	21.5%	33.7%	3.0%	< 0.001
BMI, Kg/m2	24.5 ± 3.2	24.8 ± 2.9	24.0 ± 3.6	0.016
Laboratory findings				
FBG, mmol/L	6.6 ± 1.9	6.5 ± 1.9	6.8 ± 1.9	0.062
HbA1c, mmol/L	9.1 ± 2.0	8.9 ± 2.0	9.4 ± 2.1	0.046
TC, mmol/L	4.55 ± 1.25	4.48 ± 1.22	4.78 ± 1.25	0.450
TG, mmol/L	1.89 ± 1.46	1.94 ± 1.46	1.82 ± 1.48	0.002
HDL-c, mmol/L	1.06 ± 0.29	0.98 ± 0.26	1.16 ± 0.30	< 0.001
LDL-c, mmol/L	2.76 ± 1.02	2.66 ± 1.00	2.91 ± 1.03	0.022
Albumin, g/L	40.0 ± 3.4	40.3 ± 3.4	39.6 ± 3.5	0.084
Creatinine, µmol/L	62.7 ± 31.2	69.5 ± 30.8	52.3 ± 28.9	< 0.001
eGFR, ml/min/1.73m^2^				
Cystatin C, mg/L	0.99 ± 0.34	1.02 ± 0.33	0.95 ± 0.34	0.023
CCR	7.6 ± 1.6	8.2 ± 1.6	6.7 ± 1.3	< 0.001
Serum uric, µmmol/L	328.3 ± 92.7	349.3 ± 87.0	298.7 ± 92.7	< 0.001
PTH, pg/ml	38.6 ± 15.9	40.1 ± 16.6	36.4 ± 14.7	0.020
PINP, ng/ml	40.8 ± 19.5	37.8 ± 17.7	45.4 ± 21.1	< 0.001
β-CTX, ng/ml	0.46 ± 0.24	0.43 ± 0.22	0.50 ± 0.27	0.004
25(OH)D, ng/ml	22.55 ± 8.01	23.43 ± 8.34	21.20 ± 7.30	0.006
Calcium, mmol/L	2.24 ± 0.11	2.24 ± 0.11	2.25 ± 0.11	0.593
ASM/height^2^, g/cm^2^	6.68 ± 1.26	7.18 ± 1.13	5.91 ± 1.05	< 0.001
BMD				
Total lumbar, g/cm^2^	1.122 ± 0.206	1.194 ± 0.184	1.012 ± 0.187	< 0.001
Femur neck, g/cm^2^	0.896 ± 0.149	0.941 ± 0.142	0.826 ± 0.131	< 0.001
Total hip, g/cm^2^	0.966 ± 0.151	1.007 ± 0.141	0.903 ± 0.144	< 0.001
Osteoporosis	20.6%	11.1%	34.9%	< 0.001

Values are mean SD or number (%)

*P* < 0.05 was deemed significant (comparison between men and women group)

*BMI* Body mass index, *FBG* Fasting blood glucose, *HbA1c* Glycosylated hemoglobin, *TC* Total cholesterol, *TG* Triglyceride, *HDL-c* High density lipoprotein cholesterol, *LDL-c* Low density lipoprotein cholesterol, *PTH* Parathyroid hormone, *25(OH)D* 25-hydroxy-vitamin, *CCR* Creatinine-to-Cystatin C ratio

### Comparison of CCR, and other biochemical and clinical indicators in T2DM patients with normal BMD values, osteoporosis and osteopenia

Based on the BMD T score measured by DXA, T2DM patients were divided into normal BMD, osteoporosis and osteopenia groups (Table 2). Compared with normal BMD group, BMI, albumin, creatinine, uric and ASM/height^2^ were significantly reduced and age, diabetic duration, FBG, cystatin C, PINP and β-CTX levels significantly increased in the osteoporosis and osteopenia groups. The BMD of the total hip, femur neck and total lumbar spine in the osteoporosis and osteopenia groups were significantly decreased. Compared with the normal BMD group, the level of CCR in osteoporosis group was lower.

**Table 2:** Comparison of various parameters of T2DM patients with differently BMD T value

	Normal (*n* = 193)	Osteopenia (*n* = 140)	Osteoporosis (*n* = 85)
Age, years	58.4 ± 6.6	61.8 ± 7.0^*^	64.5 ± 7.2^*#^
Diabetic duration, years	8.2 ± 6.9	9.6 ± 7.1^*^	10.7 ± 9.2^*^
Systolic blood pressure, mmHg	140.7 ± 25.0	145.5 ± 25.2	142.8 ± 25.0
Diastolic blood pressure, mmHg	84.1 ± 8.6	88.6 ± 71.3	82.3 ± 6.9
BMI, Kg/m^2^	25.2 ± 3.2	24.0 ± 3.0^*^	23.5 ± 3.3^*^
**Laboratory findings**			
FBG, mmol/L	6.4 ± 1.7	6.7 ± 2.0^*^	6.7 ± 2.1^*^
HbA1c, mmol/L	9.1 ± 2.1	9.1 ± 1.9	9.1 ± 2.1
TC, mmol/L	4.5 ± 1.3	4.5 ± 1.2	4.7 ± 1.3
TG, mmol/L	2.0 ± 1.8	2.0 ± 1.3	1.6 ± 0.9^*#^
HDL-c, mmol/L	1.0 ± 1.3	1.1 ± 0.3	1.1 ± 0.3^*#^
LDL-c, mmol/L	2.7 ± 1.0	2.7 ± 1.0	3.0 ± 1.1
Albumin, g/L	40.5 ± 3.3	40.3 ± 3.5	38.6 ± 3.2^*#^
Creatinine, µmol/L	65.9 ± 34.1	62.9 ± 27.0^*^	55.0 ± 29.8^*#^
Cystatin C, mg/L	0.96 ± 0.34	1.02 ± 0.32^*^	1.04 ± 0.33^*^
CCR,	8.3 ± 1.6	7.4 ± 1.3^*^	6.3 ± 1.3^*#^
Uric, µmmol/L	346.3 ± 91.7	325.1 ± 93^*^	297.9 ± 86.5^*#^
PTH, pg/ml	36.9 ± 14.7	38.9 ± 15.6	39.1 ± 16.7
PINP, ng/ml	37.4 ± 17.7	41.9 ± 17.6^*^	46.7 ± 24.4^*^
β-CTX, ng/ml	0.41 ± 0.20	0.47 ± 0.25^*^	0.53 ± 0.29^*^
25(OH)D, ng/ml	22.9 ± 7.6	22.9 ± 8.9	21.0 ± 7.3^*^
Calcium, mmol/L	2.25 ± 0.11	2.24 ± 0.10	2.24 ± 0.11^*^
ASM/height^2^, Kg/m^2^	7.1 ± 1.3	6.4 ± 0.9^*^	6.2 ± 1.3^*#^
**BMD**			
Total lumbar, g/cm^2^	1.257 ± 0.157	1.051 ± 0.147^*^	0.932 ± 0.176^*#^
Femur neck, g/cm^2^	1.003 ± 0.110	0.826 ± 0.092^*^	0.766 ± 0.128^*#^
Total hip, g/cm^2^	1.070 ± 0.112	0.899 ± 0.106^*^	0.839 ± 0.130^*#^

Values are mean SD or number (%)

^*^Refers to patients with normal, *P* < 0.05

^#^Refers to patients with osteopenia, *P* < 0.05

*BMI* Body mass index, *FBG* Fasting blood glucose, *HbA1c* Glycosylated hemoglobin, *TC* Total cholesterol, *TG* Triglyceride, *HDL-c* High density lipoprotein cholesterol, *LDL-c* Low density lipoprotein cholesterol, *PTH* Parathyroid hormone, *25(OH)D* 25-hydroxy-vitamin, *CCR* Creatinine-to-Cystatin C ratio

### Spearman correlations analysis between CCR, BMD and bone metabolism index

The Spearman correlation coefficients (r) of CCR and BMD of lumbar, femur neck, hip, PTH, serum Ca, ASM/height^2^ were 0.381, 0.302, 0.323, -0.141, 0.181 and0.215 in male groups, respectively. However, CCR was not correlated with the BMD in female groups (Table 3).

**Table 3:** Correlation analysis between CCR, BMD and bone metabolism markers

	Male		female	
Variables	*r*	*P*	*r*	*P*
Total lumbar BMD	0.381	< 0.001	0.107	0.192
Total hip BMD	0.302	< 0.001	0.013	0.669
Femur neck BMD	0.323	< 0.001	0.029	0.721
PTH	-0.141	0.033	-0.137	0.039
PINP	-0.087	0.194	-0.014	0.835
β-CTX	-0.026	0.693	-0.015	0.822
25(OH)D	0.050	0.451	-0.010	0.877
Calcium	0.181	0.008	0.226	0.001
ASM/height^2^	0.215	0.002	0.085	0.335

### Linear regression analyses for BMD

Age, BMI and HbA1c had a significant effect on osteoporosis prevalence. Accordingly, subgroup analysis was performed based on BMI, age and HbA1c (Table 4). After adjusting age, diabetic duration, BMI, smoking, drinking, TC, TG, HDL-C, LDL-C, albumin, uric, HbA1c and FBG, CCR had an independent relationship with the BMD of the femoral neck, lumbar spine, and hip in the male group, but that among female patients was not significant.

**Table 4:** Multivariate regression for BMD

	Total lumbar		Femur neck		Total hip	
	β	*P*	β	*P*	β	*P*
Male						
BMI < 24 kg/m^2^	0.206	0.038	0.229	0.047	0.200	0.212
BMI ≥ 24 kg/m^2^	0.271	0.015	0.252	0.044	0.212	0.049
Age < 65 years	0.285	0.012	0.269	0.020	0.271	0.020
Age ≥ 65 years	0.388	0.003	0.338	0.011	0.330	0.014
HbA1c < 9.0%	0.276	0.018	0.204	0.046	0.229	0.049
HbA1c ≥ 9.0%	0.320	0.020	0.236	0.037	0.267	0.044
Female						
BMI < 24 kg/m^2^	0.034	0.843	0.058	0.773	-0.005	0.979
BMI ≥ 24 kg/m^2^	-0.029	0.819	0.238	0.052	0.231	0.072
Age < 65 years	0.084	0.655	0.150	0.414	0.101	0.577
Age ≥ 65 years	-0.072	0.510	0.226	0.070	0.228	0.047
HbA1c < 9.0%	0.050	0.749	0.139	0.396	0.176	0.293
HbA1c ≥ 9.0%	0.026	0.820	0.200	0.108	0.185	0.091

*Abbreviations*: *BMI* Body mass index, *HbA1c* Glycosylated hemoglobin

### Logistic regression analyses of male osteoporosis

Logistic regression analysis was used to test the association between CCR and osteoporosis (Table 5). Although the odds ratio decreased after adjusting age, diabetic duration, BMI, smoking, drinking, TC, TG, HDL-C, LDL-C, albumin, uric, HbA1c and FBG, the correlation between CCR and osteoporosis remained significant in male patients (odd ratio = 0.498, *P* < 0.031), but the female group did not show such relationship.

**Table 5:** Logistic regression analysis for osteoporosis

Variables	Male		Female	
	Odds Ratio	*P*	Odds Ratio	*P*
Age	1.027 (0.925–1.140)	0.620	1.133 (1.046–1.227)	0.002
Diabetic duration	0.982 (0.875–1.102)	0.758	0.961 (0.892–1.036)	0.302
BMI	0.777 (0.574–0.954)	0.045	0.909 (0.792–1.044)	0.178
Smoking	1.574(0.368–5.224)	0.539	0.000(0.000-)	1.000
Drinking	0.549 (0.161–1.930)	0.298	0.000(0.000-)	1.000
TC	0.936 (0.099–8.868)	0.954	1.757 (0.349–8.834)	0.494
TG	1.168 (0.365–3.740)	0.794	0.627 (0.265–1.484)	1.130
HDL-c	1.859 (0.057–10.350)	0.727	0.674 (0.048–9.427)	0.769
LDL-c	0.981 (0.115–8.368)	0.986	0.804 (0.166–3.898)	0.804
Albumin	0.791 (0.593–1.056)	0.111	0.905 (0.789–1.039)	0.157
Uric	0.993 (0.984–1.003)	0.183	0.998 (0.992–1.004)	0.515
HbA1c	1.109 (0.729–1.689)	0.628	0.634 (0.420–0.956)	0.030
FPG	0.873 (0.528–1.445)	0.598	1.114 (0.848–1.463)	0.437
Creatinine/ Cystatin C	0.498 (0.255–0.973)	0.031	0.755 (0.571–1.125)	0.150

*Abbreviations*: *BMI* Body mass index, *TC* Total cholesterol, *TG* Triglyceride, *HDL-c* High density lipoprotein cholesterol, *LDL-c* Low density lipoprotein cholesterol, *FBG* Fasting blood glucose, *HbA1c* Glycosylated hemoglobin

### Prognostic value of CCR

**Fig. 1:**
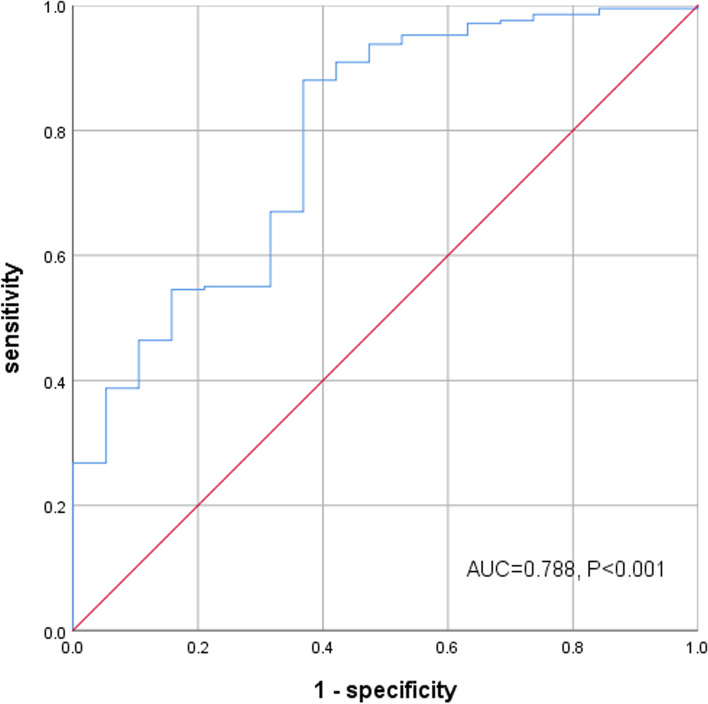
ROC curve analysis related to the impact of CCR on the osteoporosis in male group

ROC curve analysis related to the impact of CCR on the diagnosis of osteoporosis in male group (Fig. 1). The area under ROC curve was 0.788. The optimal cut-off value of CCR for predicting osteoporosis was 6.73 with the sensitivity of 88% and the specificity of 63%.

## Discussion

As far as we know, this study is the first to investigate the relationship between CCR, osteoporosis and BMD in patients in T2DM patients. The main finding of this study is that in male T2DM patients, CCR is positively correlated with BMD, at total hip, lumbar and femur neck, and negatively correlated with osteoporosis.

Reduced muscle mass, or sarcopenia, is a well-known risk factor for osteoporosis. Reduced muscle mass affects balance and thereby increases the risk of falls and subsequent fractures [14]. In this way, gradual age-related decline in bone and muscle (i.e., osteoporosis and sarcopenia) can result in increased morbidity and mortality [15]. Considering the close relationship between sarcopenia and osteoporosis and the effects of muscle mass on fracture risk, identification and treatment of those conditions is important in older populations. Therefore, recent studies have suggested a more inclusive name be given to the combination of sarcopenia and osteoporosis, such as ‘dysmobility syndrome,’ which integrates their pathogenesis and unites them as a single therapeutic target [16]. However, although osteoporosis has been clearly defined, the definition of sarcopenia remains unclear [17]. In addition, although DXA is the currently accepted gold standard test for evaluating body composition, including both bone density and muscle mass [18], it is not easily accessible or commonly available to general populations because of time and cost. From this background, we hypothesized that CCR, known to be a stable marker of sarcopenia [19], could be related to bone health status.

Serum creatinine reflects one’s physical activity status as well as skeletal muscle mass, and both are important for maintaining bone health [20]. In addition, creatinine degradation is stimulated by reactive oxygen species and in particular by the hydroxyl radical. Fernández-Real et al. reported that telomere length of subcutaneous adipose tissue cells was positively associated with serum creatinine but not with GFR. In other words, decreased serum creatinine is associated with a marker of cellular senescence and oxidative stress and consequently decreased serum creatinine may result in deterioration of BMD via oxidative stress [21].

Cystatin C is significantly associated with renal function. 1,25-dihydroxyvitamin D synthesis is decreased even in mild renal impairment, leading to reduced calcium absorption, secondary hyperparathyroidism, bone loss, and consequently, fracture [22, 23]. Taken together, we can speculate that CCR, a cheap and simple method, can be used as a marker to assess osteoporosis in type 2 diabetes and it can be also alternative for body composition analysis in subjects with normal renal function.

The association between CCR and sarcopenia has been established among the subjects with chronic obstructive pulmonary disease [24], advanced cancer [25], obstructive coronary artery disease [26] and healthy subjects [27]. Takafumi et al. recommend CCR as a practical screening marker for sarcopenia in T2DM patients [9]. Our findings are consistent with previous studies. It is found that CCR is positively associated with ASM/height^2^ in male group (*r* = 0.215, *P* = 0.002), but such relationship is not found in female group.

Reduced muscle mass can result in the worsening of insulin sensitivity, which in turn can result in diabetes [28]. Diabetes and other systemic diseases caused by sarcopenia can lead to both abnormal bone metabolism and muscle loss [29]. Our results suggest that low CCR is an important risk factor for osteoporosis and decreased BMD in male T2DM patients. As far as we know, this study is the first to show a direct correlation between CCR, osteoporosis and BMD in patients with T2DM.

The analysis of BMI, age, HbA1c subgroups further showed that among the male patients with higher BMI, older age and higher glycosylated hemoglobin levels, the association between CCR and bone mineral density was higher than that in the control group. Accordingly, for the above patients, CCR may be a simple predictor of BMD in male T2DM.

CCR is an important component of sarcopenia and a predictor of various adverse health outcomes. Lu et al. found that subjects with low CCR is a risk factor for adverse cardiovascular events in patients with obstructive coronary artery disease [9]. Liu et al. suggested that low CCR is a risk factor for long-term poor prognosis and 30-day mortality and in Acute Ischemic Stroke Patients [30]. Qiu et al. reported that low CCR can lead to increased risk of diabetes [31]. Therefore, it is valuable to identify patients with sarcopenia in clinical practice.

In our study, CCR is negatively associated with osteoporosis in male group (OR = 0.498, *P* = 0.031), but the female group does not show such relationship, Which has been confirmed in previous studies that male have a closer relationship between bone mass and muscle mass than in females [32–34]. Although we could not elucidate why this association was more prominent in men than in women, it might be explained by the fact that females tend to have lower skeletal muscle mass than males. Additionally, increased body fat in postmenopausal women might alter the bone-muscle (CCR) relationship. Conversion of androgens to estrogens in adipose tissue could have a modest effect on bone, especially in postmenopausal women [35]. However, in men, adipose tissue is not an important sex hormone source and as they have relatively small fat mass, it may not considerably influence on the bone-muscle (CCR) relationship [36].

Compared with traditional detection methods such as CT, dual-energy-X-ray absorptiometry and bioimpedance analysis, CCR detection only requires blood sampling, which is easy to be analyzed, and does not require the patients to bear the high costs of detection. As a result, CCR is more acceptable. In addition, the CCR results are easy to understand and intuitive. Therefore, it is more convenient for clinicians to use the CCR to assess osteoporosis.

This study has several limitations. First of all, it had a retrospective design, a relatively small size, and was conducted at a single hospital. Secondly, we do not know the causal relationship between osteoporosis and the CCR because of the cross-sectional design. Thirdly, we do not consider the dietary intake of meat, or drugs, such as cimetidine, determination of exercise. These factors may change creatinine levels and affect CCR. Fourthly, the concentrations of serum cystatin C and creatinine is significantly affected by renal function, suggesting that the CCR may not be suitable for identifying osteoporosis in patients with abnormal renal function, and the prevalence of osteoporosis in patients with chronic kidney diseases is relatively high [37].

## Conclusion

In summary, this study reported for the first time that lower CCR is closely related to osteoporosis and low BMD in male T2DM patients. When the diagnostic cut-off value of male CCR is 6.73, the risk of osteoporosis increase, with a sensitivity of 88% and a specificity of 63%.

## Data Availability

The data that support the findings of this study are available from Institutional Review Board of the second affiliated hospital and Yuying Children’s Hospital of Wenzhou Medical University but restrictions apply to the availability of these data, which were used under license for the current study, and so are not publicly available. Data are however available from the authors upon reasonable request and with permission of Institutional Review Board of the Second Affiliated Hospital and Yuying Children’s Hospital of Wenzhou Medical University.

## Abbreviations

CCR: Creatinine-to-cystatin C ratio
BMD: Bone Mineral Density
FBG: Fasting blood glucose
BMI: Body mass index
HbA1c: Glycosylated hemoglobin
25(OH)D: 25-Hydroxy-vitamin
PTH: Parathyroid hormone
