# Correction: Creatinine‑to‑cystatin C ratio as a marker of sarcopenia for identifying osteoporosis in male patients with type 2 diabetes mellitus

**DOI:** 10.1186/s12891-022-05707-w

**Published:** 2022-08-01

**Authors:** Huifang Dai, Jing Xu

**Affiliations:** Department of Endocrinology, The Second Affiliated Hospital and Yuying Children’s Hospital of Wenzhou Medical University, Lucheng District, Wenzhou, Zhejiang Province People’s Republic of China

**Correction: BMC Musculoskelet Disord 23, 672 (2022)**

**https://doi.org/10.1186/s12891-022-05636-8**

Following the publication of the original article [1] the first author Huifan Dai needs to be changed to Huifang Dai. The author department of Huifang Dai is the same as that of Jing Xu.

The original article [1] has been updated.
